# Generation of Connective Tissue-Free Microvascular Fragment Isolates from Subcutaneous Fat Tissue of Obese Mice

**DOI:** 10.1007/s13770-023-00571-8

**Published:** 2023-10-02

**Authors:** Friederike C. Meßner, Wolfgang Metzger, Julia E. Marschall, Caroline Bickelmann, Michael D. Menger, Matthias W. Laschke

**Affiliations:** 1 Institute for Clinical and Experimental Surgery, Saarland University, 66421 Homburg, Germany; 2 Department of Trauma, Hand and Reconstructive Surgery, Saarland University, 66421 Homburg, Germany

**Keywords:** Tissue engineering, Microvascular fragments, Obesity, Subcutaneous fat tissue, Vascularization

## Abstract

*****BACKGROUND***:**

Microvascular fragment (MVF) isolates are generated by short-term enzymatic digestion of adipose tissue and contain numerous vessel segments for the vascularization of tissue defects. Recent findings indicate that the functionality of these isolates is determined by the quality of the fat source. Therefore, we compared MVF isolates from subcutaneous adipose tissue of obese and lean mice.

*****METHODS***:**

MVF isolates were generated from subcutaneous adipose tissue of donor mice, which received a high fat or control diet for 12 weeks. The isolates were analyzed *in vitro* and *in vivo*.

*****RESULTS***:**

Feeding of mice with a high fat diet induced obesity with adipocyte hypertrophy, resulting in a significantly lower collagen fraction and microvessel density within the subcutaneous fat depots when compared to lean controls. Accordingly, MVF isolates from obese mice also contained a reduced number of MVF per mL adipose tissue. However, these MVF tended to be longer and, in contrast to MVF from lean mice, were not contaminated with collagen fibers. Hence, they could be freely seeded onto collagen-glycosaminoglycan scaffolds, whereas MVF from lean controls were trapped in between large amounts of collagen fibers that clogged the pores of the scaffolds. In line with these results, scaffolds seeded with MVF isolates from obese mice exhibited a significantly improved *in vivo* vascularization after implantation into full-thickness skin defects.

*****CONCLUSION***:**

Subcutaneous adipose tissue from obese mice facilitates the generation of connective tissue-free MVF isolates. Translated to clinical conditions, these findings suggest that particularly obese patients may benefit from MVF-based vascularization strategies.

## Introduction

Microvascular fragments (MVF) are intact vessel segments, which result from the mechanical dissection and short-term enzymatic digestion of adipose tissue [1, 2]. In microcirculation research, these vessel segments have been used for many years to investigate the function of endothelial cells and pericytes as well as basic angiogenic mechanisms and the organization of newly developing microvascular networks in two- and three-dimensional experimental settings [3–6]. More recently, MVF have also been suggested as potent vascularization units for various applications in the field of tissue engineering [7]. As such, they are seeded onto different types of biomaterials or incorporated into artificial tissue constructs [8–11], where they rapidly reassemble into new microvascular networks and develop interconnections to the surrounding host microvasculature after implantation into a tissue defect [12–14].

Besides their high vascularization capacity, MVF isolates additionally exhibit several promising properties, which may further contribute to effective tissue regeneration [15]. They are a rich source of multipotent mesenchymal stem cells that can serve as the basis for the development of specific tissue types [16–18]. Moreover, they comprise lymphatic vessel fragments and express lymphangiogenic factors, promoting the formation of new lymphatic networks [19]. Finally, MVF isolates also exert immunomodulatory effects, because they contain substantial amounts of macrophages, which can shift from a pro-inflammatory M1 polarized to a pro-angiogenic M2 polarized phenotype after transplantation [9, 20].

Recent findings indicate that the functionality of MVF isolates is determined by the origin and quality of the used fat source. For instance, we found that implanted collagen-glycosaminoglycan (CGAG) scaffolds, which are seeded with MVF isolates from the subcutaneous adipose tissue of donor mice, exhibit a significantly reduced *in vivo* vascularization when compared to implants seeded with MVF isolates from the epididymal fat pads of the identical animals [21]. This was mainly caused by the contamination of subcutaneous MVF isolates with high amounts of connective tissue fibers, which clogged the pores of the scaffolds and prevented the reconnection of individual MVF. It may be speculated that this problem particularly arises in case of the relatively small subcutaneous fat pads of lean mice. In contrast, obesity may strongly change the composition of subcutaneous adipose tissue and, thus, the ratio between adipocytes, microvessels and extracellular matrix, resulting in MVF isolates with improved handling properties. In view of the future clinical perspective that autologous MVF may be easiest isolated from liposuctioned subcutaneous fat tissue of obese patients, we tested this hypothesis in the present experimental study.

For this purpose, we first compared the histomorphology of subcutaneous adipose tissue samples from obese and lean mice. Moreover, we isolated MVF from these samples to assess their number, length distribution, viability and morphology. In addition, MVF isolates from obese and lean donor mice were seeded onto CGAG scaffolds, which were implanted into full-thickness skin defects within dorsal skinfold chambers of recipient mice to analyze their vascularization and incorporation throughout an observation period of 14 days.

## Materials and methods

### Animals and experimental diets

All animal experiments were approved by the local authorities (Landesamt für Verbraucherschutz, Saarbrücken, Germany) and conducted in accordance with the European legislation on the protection of animals (Directive 2010/63/EU) and the National Institutes of Health (NIH) guidelines on the care and use of laboratory animals (NIH publication #85–23 Rev. 1985).

For the harvesting of subcutaneous adipose tissue, we used female green fluorescent protein (GFP)^+^ donor mice (C57BL/6-Tg(CAG-EGFP)1Osb/J; The Jackson Laboratory, Bar Harbor, ME, USA) with an age of 7–10 months. In these animals, all tissues except erythrocytes and hair exhibit a strong GFP fluorescence [22]. The mice had either free access to a purified high fat diet (DIO—60 kJ% fat (Lard); ssniff Spezialdiäten GmbH, Soest, Germany) or a low fat diet (DIO –10 kJ% fat, ~ 7% sucrose; ssniff Spezialdiäten GmbH) as well as fresh water for 12 weeks. For the implantation of dorsal skinfold chambers, we used female wild-type C57BL/6 J mice (Institute for Clinical and Experimental Surgery, Saarland University, Homburg, Germany) with an age of 3–8 months. They were fed with standard pellet food (Altromin, Lage, Germany) and fresh water ad libitum. The animals were housed on wood chips as bedding under a 12 h day/night cycle.

### Harvesting of subcutaneous adipose tissue

After 12 weeks of diet, lean and obese GFP^+^ donor mice were anesthetized by an intraperitoneal injection of ketamine (75 mg/kg body weight; Ursotamin®; Serumwerke Bernburg, Bernburg, Germany) and xylazine (15 mg/kg body weight; Rompun®; Bayer AG, Leverkusen, Germany). Subsequently, the animals were euthanized by cervical dislocation and positioned under a stereomicroscope (Leica M651, Wetzlar, Germany). After thorough disinfection of the dorsal and ventral external surfaces with 70% ethanol and removal of the skin with microsurgical instruments, the entire subcutaneous adipose tissue was carefully excised, pooled, weighed and temporarily stored in a culture dish filled with Dulbecco’s modified eagle medium (DMEM; 10% fetal calf serum (FCS), 100 U/mL penicillin, 0.1 mg/mL streptomycin; Biochrom, Berlin, Germany) before further processing.

### Isolation of MVF

The subcutaneous adipose tissue was washed three times in Hanks’ Balanced Salt Solution (HBSS) without magnesium and calcium, mechanically minced by means of a scissors and enzymatically digested with collagenase type IA-S (5 U/mL; Sigma-Aldrich, Taufkirchen, Germany) under slow stirring for 7 min in an incubator at 37 °C and humidified atmospheric conditions with 5% CO_2_. During this procedure, small fractions (10 µL) of the digested tissue were repeatedly analyzed under a microscope to stop the digestion at a time point when the digestate mainly contained free and intact MVF. The digestion was neutralized with DMEM supplemented with 10% FCS and the cell-vessel suspension was incubated for 5 min at 37 °C. The fat supernatant was removed and the remaining suspension, which contained both MVF and single cells, was filtered through a 500-µm mesh to remove larger undigested tissue debris, but not MVF exhibiting a long length. Thereafter, the MVF isolate was enriched to a pellet by a 5-min centrifugation at 120 × g. The pellet was either used for *in vitro* analyses or resuspended in 10 µL 0.9% NaCl for the seeding of CGAG scaffolds.

### Number, length distribution and viability of isolated MVF

The number of individual MVF per mouse and mL adipose tissue was determined by means of a counting chamber directly after the isolation procedure. Moreover, the length distribution of MVF was analyzed under a BZ-X810 microscope (Keyence Deutschland GmbH, Neu-Isenburg, Germany). In addition, freshly isolated MVF were incubated for 15 min in 1 mL DMEM (10% FCS) containing 2 mg/mL Hoechst 33342 for nuclear staining and 1 mg/mL propidium iodide (PI) (Sigma-Aldrich) to assess the percentage of PI^+^ necrotic cells in relation to all counted cells by means of fluorescence microscopy (BZ-X810).

### Seeding of CGAG scaffolds

For scanning electron microscopy and *in vivo* analyses, MVF isolates from lean and obese donor animals were seeded onto CGAG scaffolds. These scaffolds were cut out of a commercially available 1.3-mm thick Integra® dermal regeneration template single layer without silicone sheet (Integra Life Sciences, Ratingen, Germany) [23] using a 4-mm biopsy punch (kaiEurope GmbH, Solingen, Germany). Subsequently, they were loaded in both groups with 10 µL 0.9% NaCl containing ~ 10,000 MVF.

### Scanning electron microscopy

The surface morphology of MVF-seeded CGAG scaffolds was analyzed by means of a FEI XL 30 ESEM FEG scanning electron microscope (FEI, Hillsboro, OR, USA), as described previously in detail [21].

### Dorsal skinfold chamber model

MVF-seeded CGAG scaffolds were implanted into full-thickness skin defects within a dorsal skinfold chamber [24]. The chamber consisted of two symmetrical titanium frames (Irola Industriekomponenten GmbH & Co. KG, Schonach, Germany), which were fixed on the extended dorsal skinfold of C57BL/6 J wild-type mice, as previously described in detail [25]. For this purpose, the animals were anesthetized by an intraperitoneal injection of ketamine (75 mg/kg body weight; Ursotamin®; Serumwerke Bernburg) and xylazine (15 mg/kg body weight; Rompun®; Bayer AG) and received carprofen (5 mg/kg body weight; Rimadyl®, Zoetis Deutschland GmbH, Berlin, Germany) for analgesia. After a recovery period of 48 h, the mice were anesthetized again and a dermal biopsy punch (kaiEurope GmbH) and micro scissors were used to generate a central full-thickness skin defect (diameter: 4 mm) inside the observation window of the dorsal skinfold chamber. Then, an MVF-seeded CGAG scaffold was carefully implanted into the defect and the observation window of the chamber was closed with a removable cover glass, avoiding air inclusions.

### Intravital fluorescence microscopy

Implanted MVF-seeded CGAG scaffolds were repeatedly imaged by intravital fluorescence microscopy. For this purpose, 0.1 mL of the blood plasma marker 5% fluorescein isothiocyanate (FITC)-labeled dextran (150,000 Da; Sigma-Aldrich) was injected into the retrobulbar venous plexus of the anesthetized animals for contrast enhancement. The observation window of the dorsal skinfold chamber was placed under a Zeiss Axiotech microscope (Zeiss, Oberkochen, Germany) and the microscopic images were recorded by a charge-coupled device video camera (FK6990; Pieper, Schwerte, Germany) and a DVD system.

After the *in vivo* experiments, the images were analyzed by means of the off-line analysis system CapImage (Dr. Zeintl, Heidelberg, Germany). The vascularization of the implanted scaffolds was assessed in 12 surface areas. Perfused scaffold areas (in % of all areas) were defined as areas exhibiting red blood cell (RBC)-perfused microvessels [21]. Within these areas, the functional microvessel density was determined as the total length of all RBC-perfused microvessels per area (given in cm/cm^2^). In addition, the diameter (d, given in µm) and centerline RBC velocity (v, given in µm/s) of 40 randomly selected microvessels were measured. These two parameters were used to calculate the wall shear rate (y, given in s^−1^) using the formula y = 8 × v/d [21].

### Experimental protocol

A total number of 20 GFP^+^ donor mice was randomly assigned to two groups, which were either fed with a high fat diet (obese mice; n = 10) or low fat diet (lean mice; n = 10). After 12 weeks, the animals were sacrificed and their subcutaneous adipose tissue was analyzed by histology and immunohistochemistry (n = 5 per group) and served for the generation of MVF isolates (n = 10 per group). These isolates were used to study *in vitro* the viability and length distribution of MVF (n = 5 per group) and to seed CGAG scaffolds (n = 5 per group) for scanning electron microscopic analyses. Additional MVF-seeded CGAG scaffolds (n = 10 per group) were implanted into full-thickness skin defects within dorsal skinfold chambers of GFP^−^ recipient mice (n = 10 per group) to assess their vascularization and incorporation by means of intravital fluorescence microscopy on day 0 (day of implantation), 3, 6, 10 and 14. At the end of the *in vivo* experiments, the animals were sacrificed by cervical dislocation and the dorsal skinfold chamber preparations were processed for further histological and immunohistochemical analyses.

#### Histology and immunohistochemistry

Formalin-fixed tissue specimens of subcutaneous adipose tissue from lean and obese donor mice and dorsal skinfold chamber preparations with implanted MVF-seeded CGAG scaffolds were embedded in paraffin and cut into 3 µm-thick sections. Hematoxylin and eosin (HE) stainings of individual sections were performed according to standard procedures. These sections served for the assessment of the adipocyte diameter (given in µm) and density (given in mm^−2^) in 5 randomly selected regions of interest (ROIs) within subcutaneous fat samples using a BX60 microscope (Olympus, Hamburg, Germany) and the imaging software cellSens Dimension 1.11 (Olympus). Moreover, the density of infiltrating cells (given in cells/mm^2^) was assessed in 2 ROIs in the border zones and 2 ROIs in the center of each implanted MVF-seeded CGAG scaffold. Additional sections were stained with Sirius red to measure the collagen content within both subcutaneous adipose tissue and MVF-seeded CGAG scaffolds inside dorsal skinfold chambers, as described previously in detail [13].

For immunohistochemistry, sections were co-stained with a monoclonal rat anti-mouse antibody against CD31 (1:100; Dianova, Hamburg, Germany) and a polyclonal goat antibody against GFP (1:200; Rockland Immunochemicals, Limerick, PA, USA). A goat anti-rat IgG Alexa555 antibody (Life Technologies, Ober-Olm, Germany) and a biotinylated donkey anti-goat antibody (1:30; Dianova) were used as secondary antibodies. The biotinylated antibody was detected by streptavidin-Alexa488 (1:50; Life Technologies) and cell nuclei were stained with Hoechst 33342 (2 µg/mL; Sigma-Aldrich). These stainings were used to analyze the density of CD31^+^ microvessels (given in mm^−2^) within subcutaneous adipose tissue as well as implanted MVF-seeded CGAG scaffolds and to assess the fraction of CD31^+^/GFP^+^ microvessels (given in %).

#### Statistical analysis

The data were first tested for normal distribution and equal variance. In case of parametric data, differences between the two groups were analyzed by an unpaired Student´s t-test (SigmaPlot 11.0; Jandel Corporation, San Rafael, CA, USA). In case of non-parametric data, a Mann–Whitney rank sum test was used. All values are expressed as mean ± standard error of the mean (SEM). Statistical significance was accepted for a value of *p* < 0.05.

## Results

### Histomorphology of subcutaneous adipose tissue

**Fig. 1:**
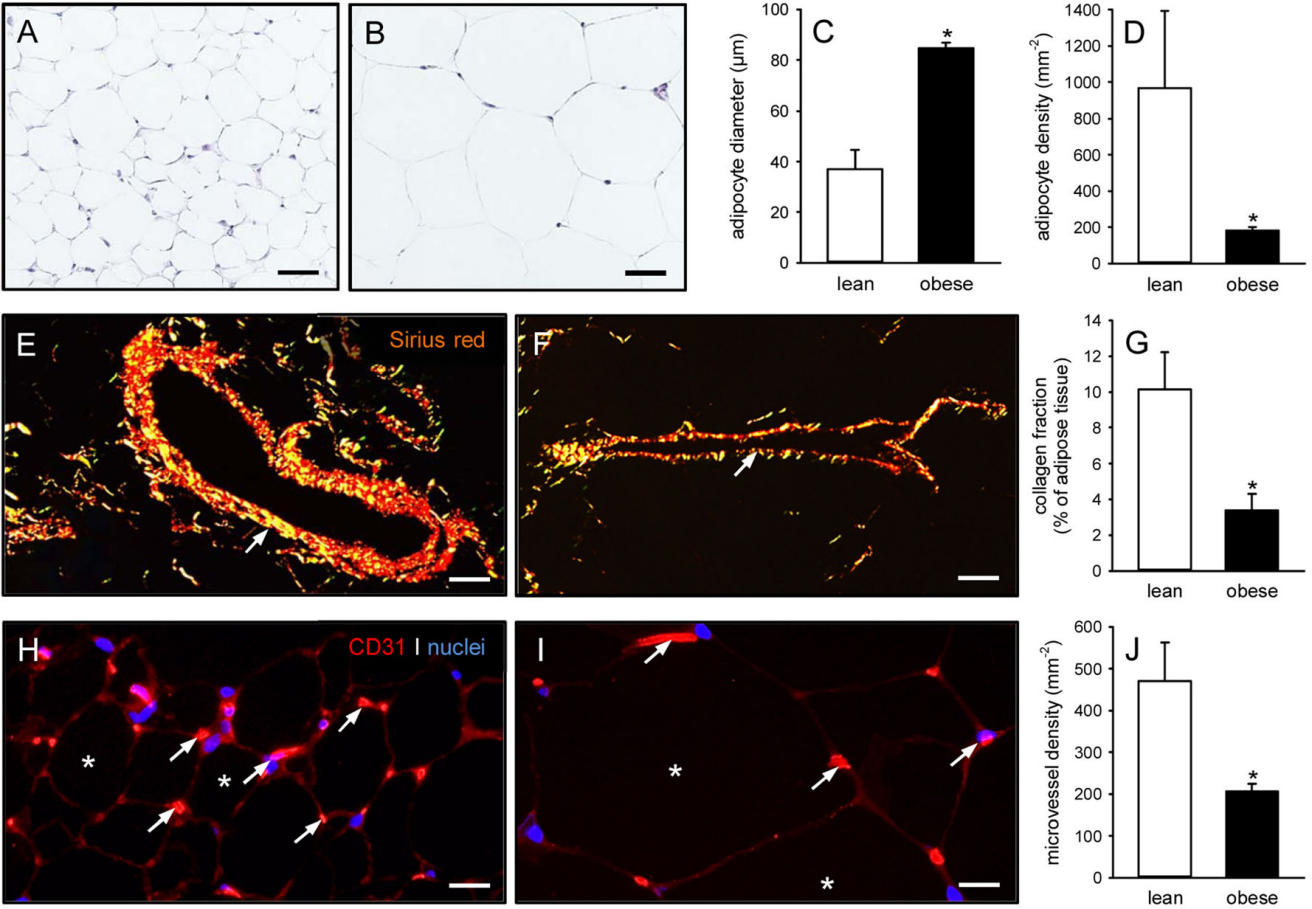
Histomorphology of subcutaneous adipose tissue from lean and obese mice. **A**, **B** HE-stained sections of the subcutaneous adipose tissue from a lean (**A**) and an obese (**B**) mouse. Scale bars: 30 µm. **C**, **D** Adipocyte diameter (**C**, given in µm) and adipocyte density (**D**, given in mm^−2^) within the subcutaneous adipose tissue from lean (white bars, n = 5) and obese (black bars, n = 5) mice. Means ± SEM. *p < 0.05 versus lean. **E**, **F** Sirius red-stained sections under polarized light, displaying larger blood vessels (arrows) with surrounding type I collagen within the subcutaneous adipose tissue from a lean (**E**) and an obese (**F**) mouse Scale bars: 40 µm. **G** Collagen fraction (given in % of adipose tissue) within the subcutaneous adipose tissue from lean (white bar, n = 5) and obese (black bar, n = 5) mice. Means ± SEM. *p < 0.05 vs. lean. **H**, **I** Immunohistochemical detection of CD31^+^ microvessels (arrows) between adipocytes (asterisks) within the subcutaneous adipose tissue from a lean (**H**) and an obese (**I**) mouse. Cell nuclei were stained with Hoechst 33342. Scale bars: 15 µm. **J** Microvessel density (given in mm^−2^) within the subcutaneous adipose tissue from lean (white bar, n = 5) and obese (black bar, n = 5) mice. Means ± SEM. *p < 0.05 versus lean

The feeding with a purified high fat diet effectively induced obesity in GFP^+^ C57BL/6 J mice, as indicated by a significantly higher body weight of 34.7 ± 2.1 g when compared to lean control animals (23.3 ± 0.4 g), which received a low fat diet over 12 weeks. Accordingly, obese mice also exhibited larger amounts of subcutaneous adipose tissue (2.8 ± 0.4 mL vs. 0.6 ± 0.1 mL; *p* < 0.05). The histomorphological analysis of this tissue revealed adipocyte hypertrophy with significantly increased adipocyte diameters and, thus, a markedly reduced adipocyte density when compared to controls (Fig. 1A, B, C, D). In consequence, the subcutaneous adipose tissue of obese mice also presented with a lower collagen fraction (Fig. 1E, F, G) and microvessel density (Fig. 1H, I, J).

### Characterization of MVF isolates

**Fig. 2:**
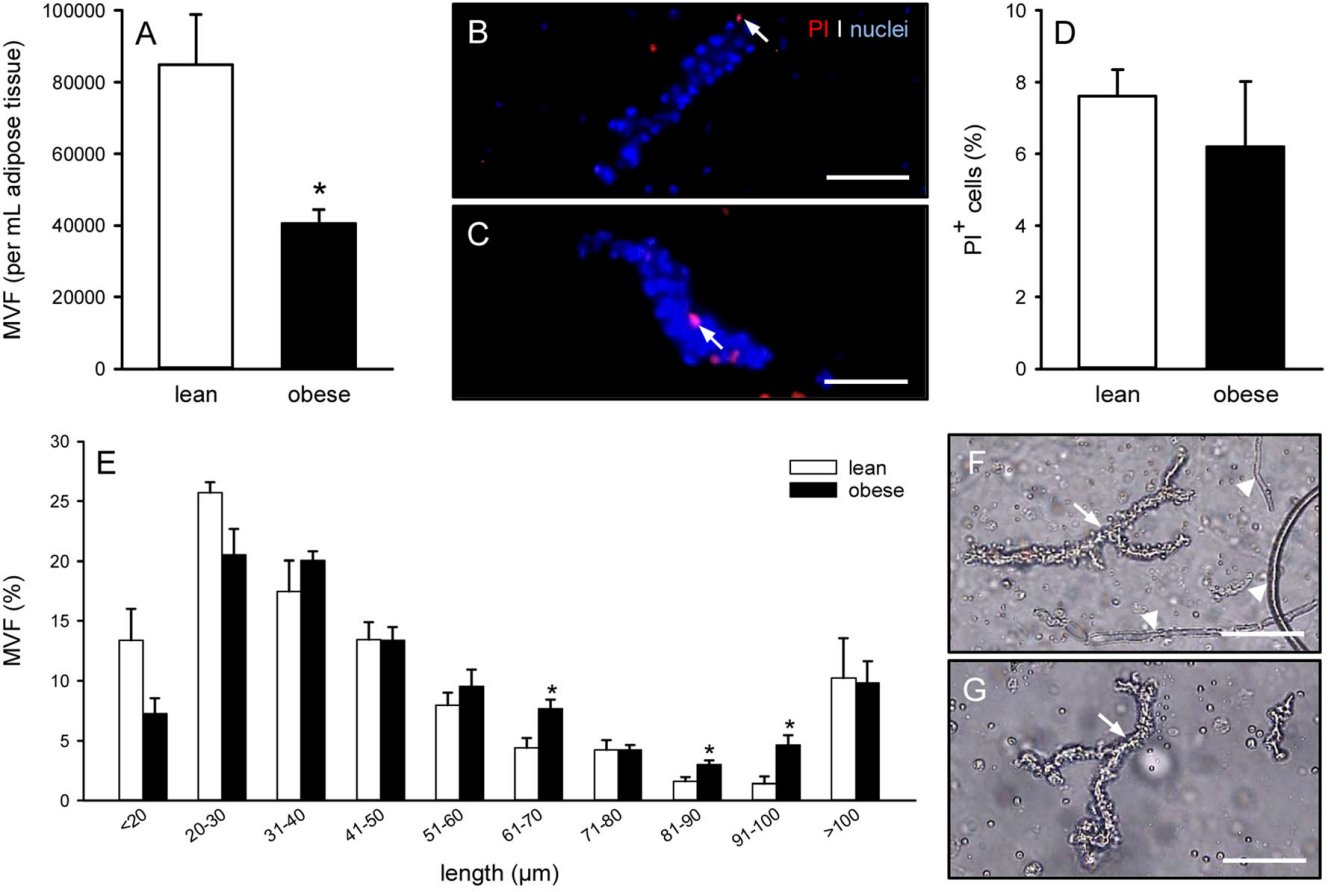
Characterization of MVF isolates. **A** Number of MVF (given per mL adipose tissue) isolated from the subcutaneous adipose tissue of lean (white bar, n = 10) and obese (black bar, n = 10) mice. Means ± SEM. *p < 0.05 versus lean. **B**, **C** Fluorescence microscopic images of PI-stained MVF isolated from the subcutaneous adipose tissue of a lean (**B**) and an obese (**C**) mouse (arrows = dead PI^+^ cells). Cell nuclei were stained with Hoechst 33342. Scale bars: 45 μm. **D** PI^+^ cells (given in % of all counted cells) within MVF isolated from the subcutaneous adipose tissue of lean (white bar, n = 5) and obese (black bar, n = 5) mice. Means ± SEM. **E** Length distribution (given in %) of MVF isolated from the subcutaneous adipose tissue of lean (white bars, n = 5) and obese (black bars, n = 5) mice. Means ± SEM. *p < 0.05 versus lean. **F**, **G** Light microscopic images of MVF isolates from the subcutaneous adipose tissue of a lean (**F**) and an obese (**G**) mouse (arrows = MVF; arrowheads = connective tissue fibers). Scale bars: 90 µm

Following a standardized isolation protocol, it was possible to generate MVF isolates from the subcutaneous adipose tissue of lean and obese mice without differences in the digestion time. As expected, a higher number of MVF per mouse could be isolated from obese mice when compared to lean control animals (119,000 ± 22,000 vs. 46,000 ± 7000; *p* = 0.054). However, in line with our histomorphological analyses, the isolates from obese mice contained a significantly reduced number of MVF per mL adipose tissue (Fig. 2A). In both groups, these freshly isolated MVF exhibited a high viability, as indicated by a comparably low fraction (< 8%) of PI^+^ necrotic cells (Fig. 2B, C, D). Of interest, further analyses of the MVF length distribution revealed a tendency towards longer vessel segments in isolates from obese mice when compared to controls (Fig. 2E). Moreover, these isolates consisted almost only of MVF and surrounding single cells, whereas MVF isolates from lean mice additionally contained many connective tissue fibers (Fig. 2F, G).

### Surface morphology of MVF-seeded CGAG scaffolds

**Fig. 3:**
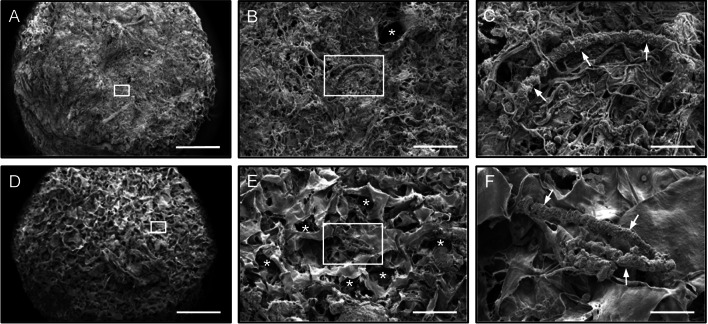
Surface morphology of MVF-seeded CGAG scaffolds. **A**–**F** Scanning electron microscopic images of CGAG scaffolds directly after their seeding with MVF isolates from the subcutaneous tissue of a lean (**A**–**C**) and an obese (**D**–**F**) MVF mouse. B and C as well as E and F display higher magnifications of white frames in A and B as well as D and E (arrows = MVF; asterisks = scaffold pores). Scale bars: A, D = 790 µm; B, E = 170 µm; C, F = 45 µm

In a next step, CGAG scaffolds were seeded with MVF isolates from lean and obese mice and analyzed by means of scanning electron microscopy. As already reported in a previous study [21], scaffolds seeded with MVF isolates from the subcutaneous adipose tissue of lean mice exhibited a markedly altered surface morphology. In fact, they were coated with a dense network of connective tissue fibers, which clogged most of the scaffold pores (Fig. 3A, B). A few MVF trapped in between these fibers could be identified (Fig. 3C). In contrast, scaffolds seeded with MVF isolates from obese mice presented with a typical morphology, as already observed in former studies using MVF isolates from the visceral, epididymal fat pads of donor mice [26, 27]. This morphology was characterized by many open scaffold pores and randomly distributed MVF freely lying on the scaffold surface (Fig. 3D, E, F).

### Vascularization of implanted MVF-seeded CGAG scaffolds

**Fig. 4:**
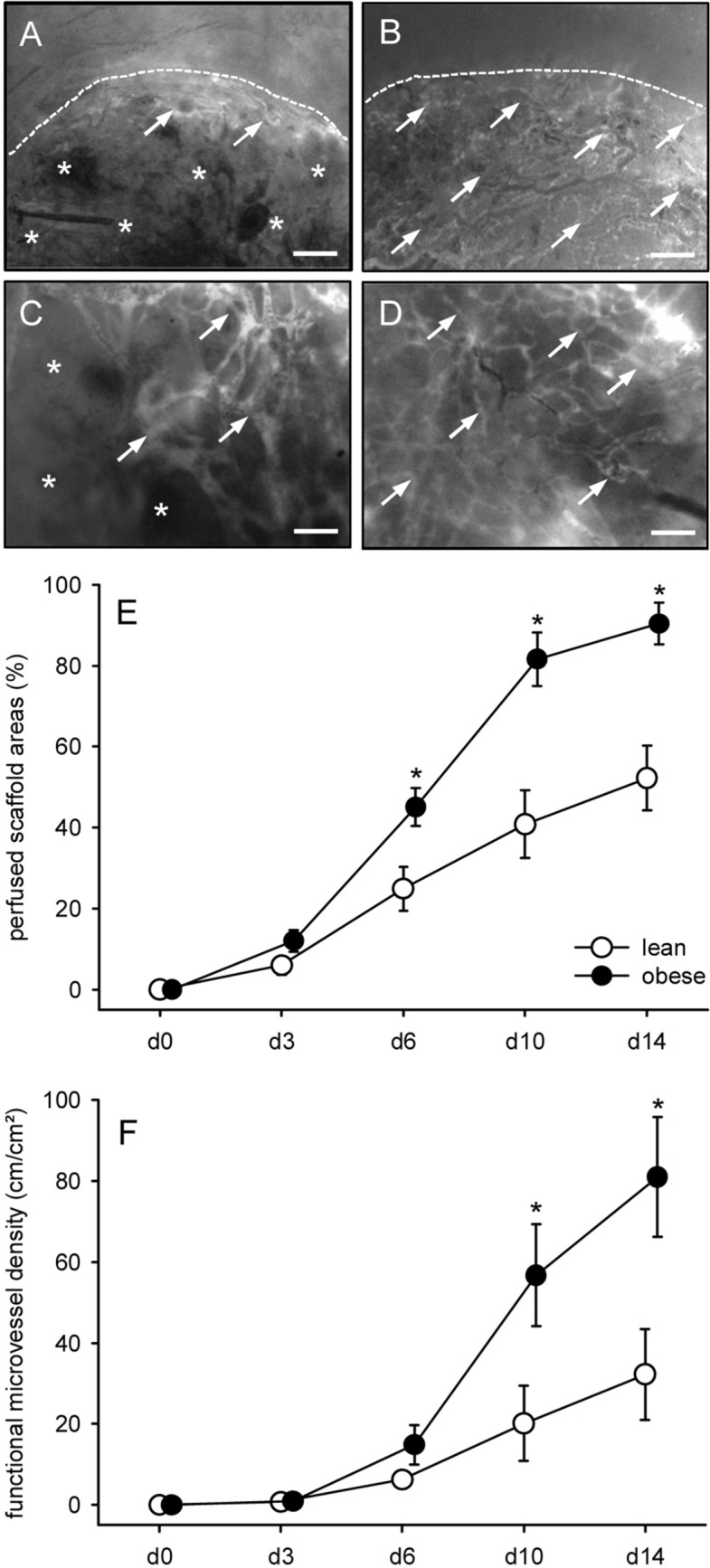
Vascularization of implanted MVF-seeded CGAG scaffolds. **A**–**D** Intravital fluorescence microscopy in blue light epi-illumination (contrast enhancement with 5% FITC-labeled dextran) of CGAG scaffolds (borders marked by broken lines in **A** and **B**) seeded with MVF isolates from the subcutaneous adipose tissue of a lean (**A**, **C**) and an obese (**B**, **D**) mouse on day 14 after implantation into full-thickness skin defects within the dorsal skinfold chamber of recipient mice (arrows = blood-perfused microvessels, asterisks = non-perfused implant areas). Scale bars: A, B = 280 µm; C, D = 60 µm. **E**, **F** Perfused scaffold areas (**E**, given in %) and functional microvessel density (**F**, given in cm/cm^2^) of CGAG scaffolds seeded with MVF isolates from the subcutaneous adipose tissue of lean (white circles, n = 10) and obese (black circles, n = 10) mice on day (d) 0, 3, 6, 10 and 14 after implantation into full-thickness skin defects within the dorsal skinfold chamber of recipient mice. Means ± SEM. *p < 0.05 versus lean

For *in vivo* analyses, CGAG scaffolds seeded with MVF isolates from GFP^+^ lean and obese mice were implanted into full-thickness skin defects within dorsal skinfold chambers of GFP^−^ recipient mice to study their vascularization by means of intravital fluorescence microscopy throughout an observation period of 14 days. These analyses revealed that the implants of both groups developed new microvascular networks over time, which interconnected with the surrounding host microvasculature, as indicated by the onset of FITC-labeled blood perfusion in individual microvessels (Fig. 4A, B, C, D, E). Of interest, this vascularization process was markedly accelerated and improved in scaffolds seeded with MVF isolates from obese mice. They exhibited a higher number of perfused scaffold areas and a higher functional microvessel density between day 6 and 14 after implantation when compared to controls (Fig. 4E, F). Accordingly, they were almost completely vascularized at the end of the *in vivo* experiment, whereas implants of the control group still exhibited large non-perfused areas on day 14 (Fig. 4A, B, C, D).

The additional assessment of microhemodynamic parameters within the implants did not show marked differences between the two groups (Table 1). As typical signs for the development and remodeling of new microvascular networks, the diameter of blood-perfused microvessels progressively decreased and the centerline RBC velocity increased in both groups over time. Accordingly, calculated values of the wall shear rate also increased until the end of the observation period without significant differences between the two groups (Table 1).

**Table 1:** Diameter (given in µm), centerline RBC velocity (given in µm/s) and wall shear rate (given in s^−1^) of individual microvessels within CGAG scaffolds seeded with MVF isolates from the subcutaneous adipose tissue of lean (n = 10) and obese (n = 10) mice on day (d) 0, 3, 6, 10 and 14 after implantation into full-thickness skin defects within the dorsal skinfold chamber of recipient mice

	0d	3d	6d	10d	14d
*Diameter (µm)*					
Lean	–	21.3 ± 1.8	29.9 ± 2.5	25.4 ± 2.1	18.8 ± 1.5
Obese	–	36.4 ± 4.4*	28.1 ± 2.1	24.3 ± 1.4	20.8 ± 1.6
*Centerline RBC velocity (µm/s)*					
Lean	–	58.2 ± 29.3	129.0 ± 19.4	127.4 ± 36.7	126.9 ± 30.6
Obese	–	26.2 ± 16.3	72.5 ± 21.2	69.7 ± 20.8	118.1 ± 31.6
*Wall shear rate (s*^*−1*^*)*					
Lean	–	26.8 ± 16.5	48.5 ± 9.4	52.4 ± 12.2	69.8 ± 16.3
Obese	–	8.3 ± 5.6	26.2 ± 7.5	30.3 ± 8.5	60.4 ± 18.4

*p < 0.05 versus lean

Mean ± SEM

### Histomorphology of implanted MVF-seeded CGAG scaffolds

**Fig. 5:**
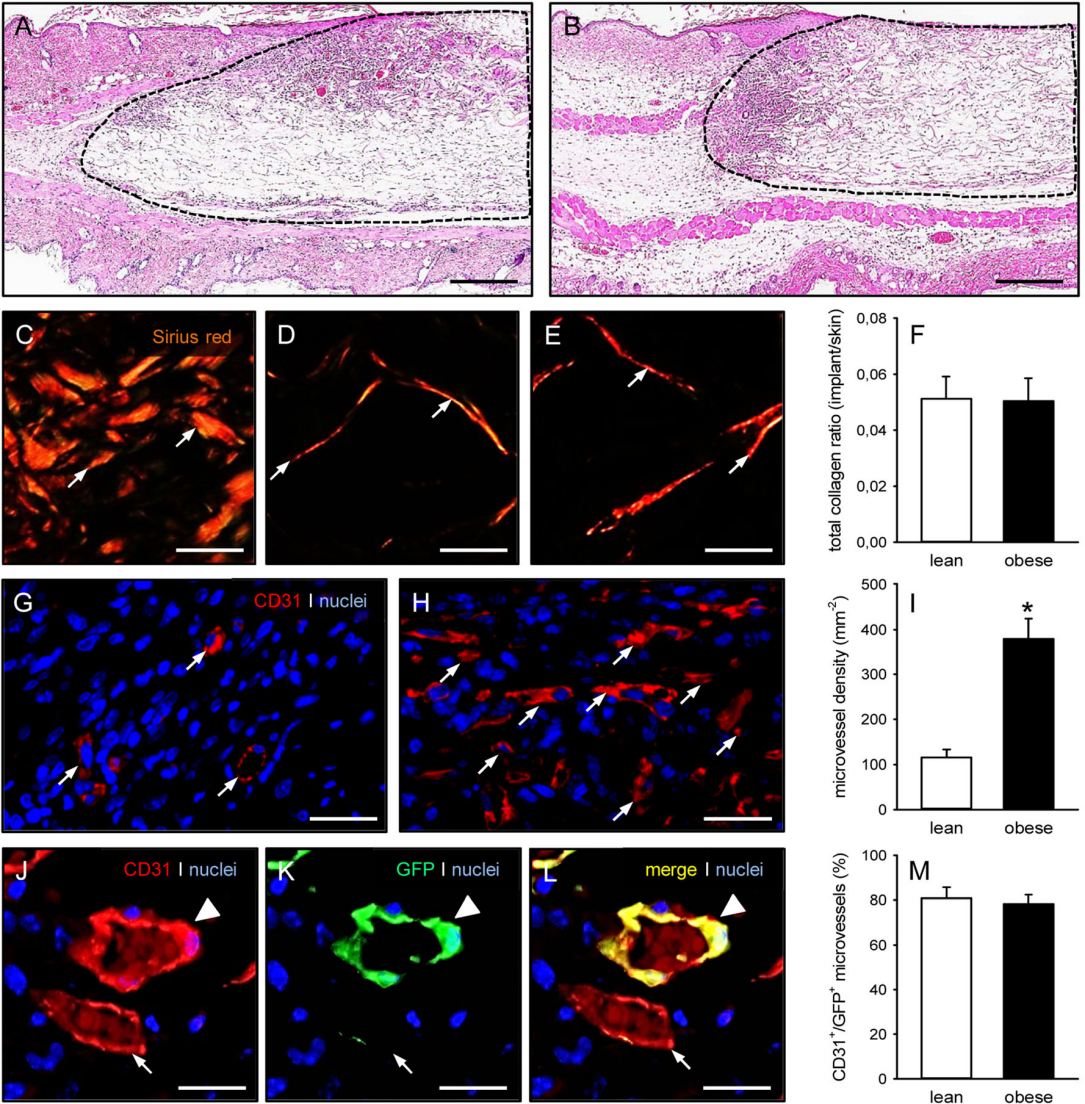
Histomorphology of implanted MVF-seeded CGAG scaffolds. **A**, **B** HE-stained sections of CGAG scaffolds (borders marked by broken lines) seeded with MVF isolates from the subcutaneous adipose tissue of a lean (**A**) and an obese (**B**) mouse on day 14 after implantation into full-thickness skin defects within the dorsal skinfold chamber of recipient mice. Scale bars: 230 µm. **C**–**E** Sirius red-stained sections under polarized light of normal skin (**C**) as well as CGAG scaffolds seeded with MVF isolates from the subcutaneous adipose tissue of a lean (**D**) and an obese (**E**) mouse (arrows = collagen fibers). Scale bars: 12 µm. **F** Total collagen ratio (given as implant/skin) of CGAG scaffolds seeded with MVF isolates from the subcutaneous adipose tissue of lean (white bar, n = 10) and obese (black bar, n = 10) mice on day 14 after implantation into full-thickness skin defects within the dorsal skinfold chamber of recipient mice. Means ± SEM. **G**, **H** Immunohistochemical detection of CD31^+^ microvessels (arrows) within CGAG scaffolds seeded with MVF isolates from the subcutaneous adipose tissue of a lean (**G**) and an obese (**H**) mouse. Cell nuclei were stained with Hoechst 33342. Scale bars: 30 µm. **I** Microvessel density (given in mm^−2^) of CGAG scaffolds seeded with MVF isolates from the subcutaneous adipose tissue of lean (white bar, n = 10) and obese (black bar, n = 10) mice on day 14 after implantation into full-thickness skin defects within the dorsal skinfold chamber of recipient mice. Means ± SEM. *p < 0.05 versus lean. **J**–**L** Immunohistochemical detection of a CD31^+^/GFP^+^ microvessel (arrowhead) and a CD31^+^/GFP^−^ microvessel (arrow) within a CGAG scaffold seeded with an MVF isolate from the subcutaneous adipose tissue of a lean mouse. Cell nuclei were stained with Hoechst 33342. Scale bars: 20 µm. **M** CD31^+^/GFP^+^ microvessels (given in %) within CGAG scaffolds seeded with MVF isolates from the subcutaneous adipose tissue of lean (white bar, n = 10) and obese (black bar, n = 10) mice on day 14 after implantation into full-thickness skin defects within the dorsal skinfold chamber of recipient mice. Means ± SEM

Additional histological analyses on day 14 revealed a comparable incorporation of implanted CGAG scaffolds seeded with MVF isolates from either lean or obese mice into the surrounding host tissue of the dorsal skinfold chamber (Fig. 5A, B). The implants did not differ in their number of infiltrating cells (lean mice: 1673 ± 500 cells/mm^2^; obese mice: 2136 ± 515 cells/mm^2^) and total collagen ratio (Fig. 5C, D, E, F). However, in line with our intravital fluorescent microscopic results, scaffolds seeded with MVF isolates from obese mice presented with a significantly higher microvessel density when compared to controls (Fig. 5G, H, I). Finally, we found that the implants of both groups contained a high fraction (~ 80%) of CD31^+^/GFP^+^ microvessels (Fig. 5J, K, L, M).

## Discussion

During the last years, an increasing number of studies indicate that adipose tissue-derived MVF are effective vascularization units for various applications in the field of regenerative medicine [28–33]. For this purpose, they may be isolated from subcutaneous adipose tissue, which can be harvested minimal-invasively by liposuction, and autologously retransferred into patients in an intra-operative one-step procedure. This future clinical scenario may be particularly feasible in obese patients with extensive subcutaneous fat depots containing sufficient amounts of MVF for the vascularization of large tissue defects. In this context, we could demonstrate in the present study that compared to lean controls the subcutaneous adipose tissue of obese donor mice facilitates the generation of connective tissue-free MVF isolates.

Under experimental conditions, MVF are typically isolated from the epididymal fat pads of mice and rats [1, 34, 35], because these prominent visceral fat depots are easy to identify and harvest. However, it is well known that visceral and subcutaneous adipose tissue markedly differs in developmental timing, molecular signature, histomorphology and biological function [36]. For instance, visceral fat exhibits a uniform structure with large unilocular adipocytes, whereas subcutaneous fat is characterized by a heterogeneous mixture of unilocular adipocytes intercalated with small multilocular adipocytes, a higher microvessel density and more extracellular matrix compounds [21, 36]. Of interest, we herein found that the histomorphology of subcutaneous fat markedly changes under diet-induced obesity into a phenotype, similar to that of visceral fat in lean mice, with a lower collagen fraction and microvessel density. This also had a significant effect on the generation of MVF isolates. In fact, isolates from the subcutaneous adipose tissue of obese donor mice contained less MVF per mL fat tissue when compared to those from lean controls. Accordingly, it may be assumed that the identical amount of fat tissue from obese donors is less effective in stimulating angiogenesis and tissue formation when compared to that of lean donors. This, however, does not represent a major problem, because obese donors also provide much larger fat depots for MVF isolation to compensate for this discrepancy. On the other hand, isolates from obese mice tended to contain longer MVF when compared to controls, which could be an advantage for their *in vivo* vascularization capacity, because longer MVF may bridge wider distances within scaffolds or tissue defects. Moreover, isolates from obese mice were almost free of connective tissue fibers. Hence, our scanning electron microscopic analyses revealed that they can be easily seeded onto CGAG scaffolds without clogging the implants’ pores.

In line with our *in vitro* results, we could further demonstrate that scaffolds seeded with MVF isolates from obese mice exhibited an improved vascularization after implantation into full-thickness skin defects of recipient animals when compared to scaffolds seeded with MVF isolates from lean control animals. Of note, for these *in vivo* analyses the scaffolds of both groups were seeded with an identical number of MVF to exclude any possibility of bias due to different numbers of isolated MVF per mL adipose tissue in lean and obese donor mice. Moreover, we used the technique of intravital fluorescence microscopy to assess the vascularization of the implants. This approach bears the major advantage that it enables the *in vivo* visualization of blood perfusion within individual microvessels [37], directly proving their functionality. For this purpose, the herein applied dorsal skinfold chamber model has already been shown in previous studies to provide highly standardized conditions for the implantation of MVF-seeded scaffolds and easy access for their repeated microscopy [14, 20, 21].

The improved *in vivo* vascularization of CGAG scaffolds seeded with MVF from obese mice was most probably caused by the aforementioned absence of undigested connective tissue fibers within the isolates. In fact, our scanning electron microscopic analyses of freshly seeded scaffolds revealed that scaffolds seeded with MVF isolates from the subcutaneous adipose tissue of lean mice are coated with a dense network of connective tissue fibers, which clog most of the scaffold pores. In contrast, scaffolds seeded with MVF isolates from obese mice were characterized by many open scaffold pores and randomly distributed MVF freely lying on the scaffold surface. On the other hand, it may be speculated that diet-induced obesity increases the intrinsic angiogenic activity of MVF. However, we did not perform further analyses in this direction, because an abundance of current literature clearly indicates that this is not the case. Indeed, obesity rather seems to be associated with an impaired angiogenesis in hypertrophic adipose tissue [38]. This may be caused by the disruption of adequate angiogenic signaling or the up-regulation of anti-angiogenic factors, such as vascular endothelial growth factor (VEGF) A165b [39, 40]. In addition, hypertrophied adipocytes have been shown to produce collagen types that inhibit the development of new blood vessels [41]. These suppressive effects on the microvasculature, in turn, likely contribute to metabolic dysfunction [42]. To overcome this problem it has been suggested that the stimulation of angiogenesis within hypertrophic adipose tissue may represent a promising therapeutic strategy in obesity [38].

At the end of our *in vivo* experiments, we additionally analyzed implanted MVF-seeded CGAG scaffolds by means of histology and immunohistochemistry. These analyses revealed that scaffolds seeded with MVF isolates from obese mice exhibited a significantly higher microvessel density on day 14 when compared to control scaffolds, which confirms our intravital fluorescence microscopic results. However, in contrast to previous studies this was not associated with a higher total collagen ratio, which is an indicator for the extent of implant incorporation [20]. This observation may be explained by the fact that scaffolds seeded with MVF isolates from lean mice were initially contaminated with much more collagen fibers. Thus, although the improved vascularization of scaffolds seeded with MVF isolates from obese mice may have promoted collagen formation over time, this effect was probably not detectable due to different collagen amounts in the two scaffold groups at the time point of *in vivo* implantation. In addition, we found that in both groups most of the microvessels within the scaffolds were GFP^+^. This clearly shows that the seeded MVF originating from GFP^+^ donor mice survived within the scaffolds and mainly contributed to their blood supply, whereas ingrowing GFP^−^ microvessels from the surrounding host tissue were of minor relevance for the vascularization of the implants.

Taken together, our novel findings demonstrate that subcutaneous adipose tissue from obese mice facilitates the generation of connective tissue-free MVF isolates when compared to that of lean control animals. If this finding holds true in humans, it indicates that particularly obese patients may benefit from MVF-based vascularization strategies in future clinical practice. Nonetheless, further optimization of the MVF isolation process may also result in MVF isolates with a reduced connective tissue contamination in lean patients. For instance, this could be achieved by additional centrifugation and filtration steps. Although this may be associated with the loss of individual MVF, it should not be a major problem considering the fact that even in lean human donors the amounts of harvestable subcutaneous adipose tissue are much higher when compared to mice.
